# Ultrasound Imaging in Sport-Related Muscle Injuries: Pitfalls and Opportunities

**DOI:** 10.3390/medicina57101040

**Published:** 2021-09-29

**Authors:** Marco Paoletta, Antimo Moretti, Sara Liguori, Francesco Snichelotto, Ilaria Menditto, Giuseppe Toro, Francesca Gimigliano, Giovanni Iolascon

**Affiliations:** 1 Department of Medical and Surgical Specialties and Dentistry, University of Campania Luigi Vanvitelli, 80138 Naples, Italy; marco.paoletta@unicampania.it (M.P.); sara.liguori@unicampania.it (S.L.); francesco.snichelotto@studenti.unicampania.it (F.S.); ilaria.menditto@studenti.unicampania.it (I.M.); giuseppe.toro@unicampania.it (G.T.); giovanni.iolascon@unicampania.it (G.I.); 2 Department of Mental and Physical Health and Preventive Medicine, University of Campania Luigi Vanvitelli, 81100 Naples, Italy; francesca.gimigliano@unicampania.it

**Keywords:** ultrasound, muscle injuries, sports, athletes, skeletal muscle, return to sport, imaging, rehabilitation

## Abstract

Muscle injuries occur frequently in athletes, accounting for more than one-third of sport-related trauma. Athletes most affected by these injuries are those practicing football and track and field, with hamstrings and gastrocnemius-soleus as the mainly involved sites. Muscle injuries lead to loss of competitions, long recovery times and risk of re-injury with a consequent increase of the management costs. It is therefore advisable to make an accurate and timely diagnosis to establish appropriate interventions for proper healing in the shortest time. In this context, ultrasound imaging is widely used for diagnosis of musculoskeletal disorders because of several advantages including absence of radiation, portability, good spatial resolution, and the ability to perform dynamic tests. The aim of this review is to address the role of US in the evaluation of athletes with muscle injuries. US may play a pivotal role for the management of sport-related muscle injuries because it is fast and relatively cheap, allowing dynamic muscle assessment and time series evaluation of the healing process.

## 1. Introduction

Sport-related muscle injury has been defined as ‘‘a traumatic distraction or overuse injury of the muscle leading to a player being unable to fully participate in training or match play’’ [1] (p. 3). Muscle injury represents more than one-third of sport-related trauma and its incidence increases with the age [1,2,3]. The risk of occurrence of this lesion is mostly observed in sports requiring maximal contractions, such as soccer and track and field. In these sports, muscle injury mainly affects biarticular muscles, in particular those with high percentage of fast-twitch fibers [2,4]. Soleus-gastrocnemius lesions are the most common muscle injuries in high-speed running, although in other sports (i.e., soccer) hamstrings and the rectus femoris are most involved [4].

Muscle lesions might cause loss of competitions and long functional recovery times [5]. Moreover, a premature return to play (RTP) may be related to high risk of recurrent injury and prolonged healing time [6]. Therefore, a timely and accurate diagnosis is required to identify the type and severity of injury to propose an appropriate management plan for complete muscle healing, reducing the risk of re-injury [7]. Magnetic Resonance Imaging (MRI) is the most sensitive technique for the detection of muscle injuries, also for minimal lesions, representing a reference standard to complete the workup of muscle injuries following physical examination. However, ultrasound (US) imaging is most used in clinical practice [8]. This cheap and non-invasive method provides an adequate characterization of muscle lesions thanks to an optimal spatial resolution. Moreover, US allows to perform dynamic assessment before and after contraction [9]. The aim of this paper is to carry out an extensive review about US imaging in sport-related muscle injuries.

## 2. Technical Aspects of Ultrasound Imaging in Skeletal Muscle Examination

Skeletal muscle is a classical target for US imaging considering it is a relatively superficial tissue. The choice of an appropriate probe for US imaging to investigate skeletal muscle depends on different factors [10]. Usually, muscle injury can be adequately visualized using high frequency linear probes (>10 MHz). In certain cases, in patients with conspicuous adipose tissue or thick muscle mass, low frequency convex probes can be used [2,11].

As the frequency of the waves increases, the spatial resolution (the ability to distinguish two separate objects) also improves. Therefore, the smaller lesions will be easier to visualize as the frequency increases but at the same time there will be a greater absorption of the waves. In this condition it will be more difficult to visualize the deep tissues. [2,12,13]. Considering advances in hardware technology and software systems, such as the use of tissue harmonic imaging, postprocessing algorithms of the returned signal or ultra-high frequency probes (up to 17 MHz), it is possible to improve the signal-to-noise ratio and tissue contrast to better visualize muscle architecture. Under optimal conditions, such as the use of high frequency probes and observation of superficial structures, it is possible to reach a spatial resolution of less than 200 microns with tissue sections of 1–0.5 mm thickness, that results even higher than the resolution of MRI [9]. In case of large lesions, extended field of view imaging (FOV) may be useful to define the real extent of the damage [9]. In a routine US exam, it has been suggested to start visualizing long and short axis on the hypothetical site of the lesion (suggested by physical examination) and then continue with slow and accurate movement of the probe on muscle belly from its origin to distal insertion. It is appropriate to evaluate enthesis, myotendinous junction, as well as epimysium and intermuscular septa that may be also affected depending on the mechanism of muscle injury. The lesion area can be dynamically detected during muscle contraction or passive mobilization to define the extent of the injury, particularly related to functional impairment [9,11].

## 3. Ultrasound Anatomy of Skeletal Muscle

**Figure 1:**
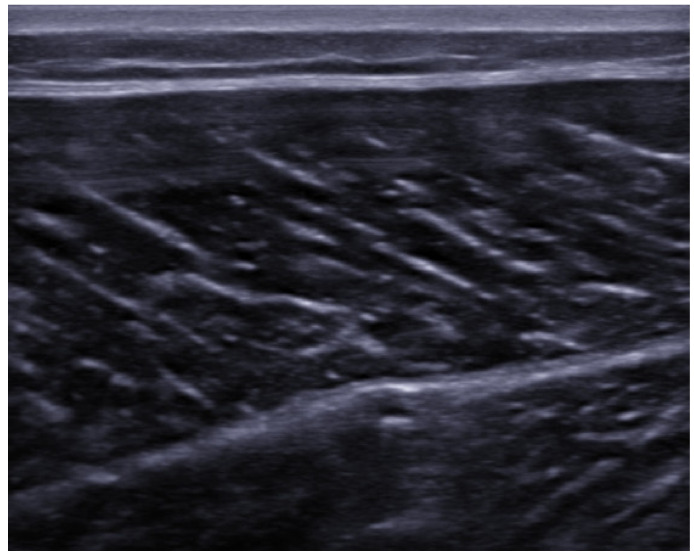
Long-axis view of the medial head of the gastrocnemius muscle in healthy individual showing normal skeletal muscle architecture.

In the context of US imaging, myofibers appear hypoechoic compared to adjacent connective and nervous structures, while fibroadipose tissues (perimysium and epimysium) are hyperechogenic. Different orientation of muscle fibers with respect to the US beam might cause artifacts of anisotropy, especially in long axis evaluation. Longitudinal axis images of muscle tissue show an alternation of parallel hypo/hyperechoic bands *(*“*veins on a leaf*”*)* with a variable orientation depending on pennation angle (Figure 1).

**Figure 2:**
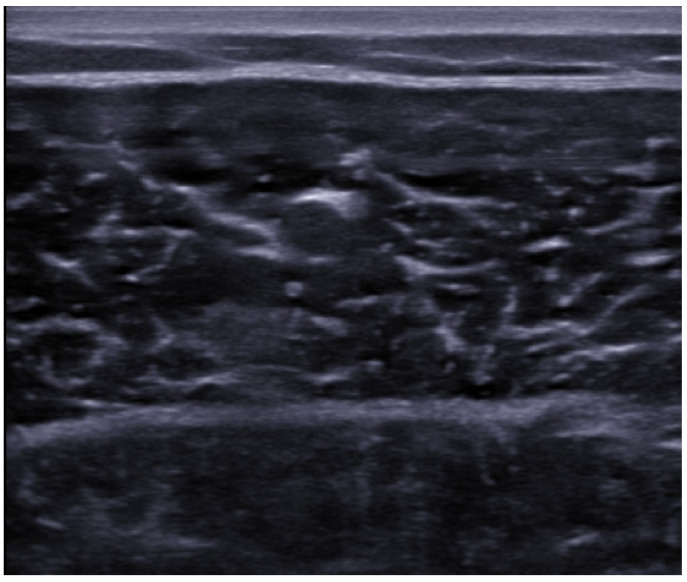
Short-axis view of the medial head of the gastrocnemius muscle in healthy individual.

In the transverse axis, the tissue appears to be formed by a background hypoechoic compound with hyperechoic dots inside *(*“*starry night*”*)* (Figure 2).

The appearance of the myotendinous junction (MTJ) will vary depending on the type of muscle, but generally it has a progressively hyperechoic appearance with respect to the muscle tissue. Tendons and the epimysium have a hyperechoic appearance, but the former can give artifacts of anisotropy due to their fibrillar structure [2,9,11].

## 4. Muscle Injuries: Types and Mechanisms

Acute muscle injuries are classified as direct if the muscle damage occurs at the site of application of external injury force or indirect if due to an internal force (e.g., mechanical stress generated by muscle contraction or stretching) [7,14,15].

Direct injuries include contusions and lacerations, in case of penetrating trauma, and are typical of contact sports such as soccer or rugby [2,16,17]. The extent of tissue damage depends on the amount of force applied. However, a muscle contraction during the impact could better absorb the force, resulting in lower damage [7,18,19]. Moreover, the size of direct muscle injuries might not correlate with clinical signs and functional impairment [5].

Direct lesions have been classified according to the extent of the clinical signs in [5,9]:

- mild form (loss of range of movement (ROM) less than one-third with short recovery time);
- moderate form (loss of ROM between one- and two-thirds with moderate recovery time);
- severe form (with loss of ROM larger than two-thirds with long recovery time).

Indirect injuries include muscle strain. The mechanism underlying this type of damage is a forced elongation of the muscle fibers, usually occurred during an eccentric contraction that exceeds viscoelastic limits of the tissue. Myotendinous junction is most involved probably due to a different tissue elasticity, which is significantly lower in the tendon component than in the muscular one [8,15]. Factors favoring the onset of indirect muscle injuries are eccentric contraction, muscles crossing two joints (e.g., hamstring, rectus femoris), high percentage of fast-twitch fibers within muscle, and muscle imbalance between agonist and antagonists resulting in failure in absorbing or dissipating applied forces [9,20,21]. In rare cases, during a strong contraction, an avulsion injury may occur with detachment of the bony surface of tendon insertion [22]. Indirect muscle injuries can be classified according to clinical criteria in:

- grade 1: no significant loss of function and strength, and minimal tissue tearing (less than 5%);
- grade 2: myotendinous junction injury with evident reduction in strength and function;
- grade 3: complete injury to the myotendinous unit and total loss of strength and function [9].

A further type of indirect muscle injury is the “delayed onset muscle soreness” (DOMS). This is typically caused by an excessive physical activity requiring eccentric contractions. In DOMS, a reduction in strength and muscle function associated with pain develops in the 24–72 h following the muscle effort, with a progressive and spontaneous slow recovery [9,23].

## 5. Ultrasound Findings of the Muscle Lesions

**Figure 3:**
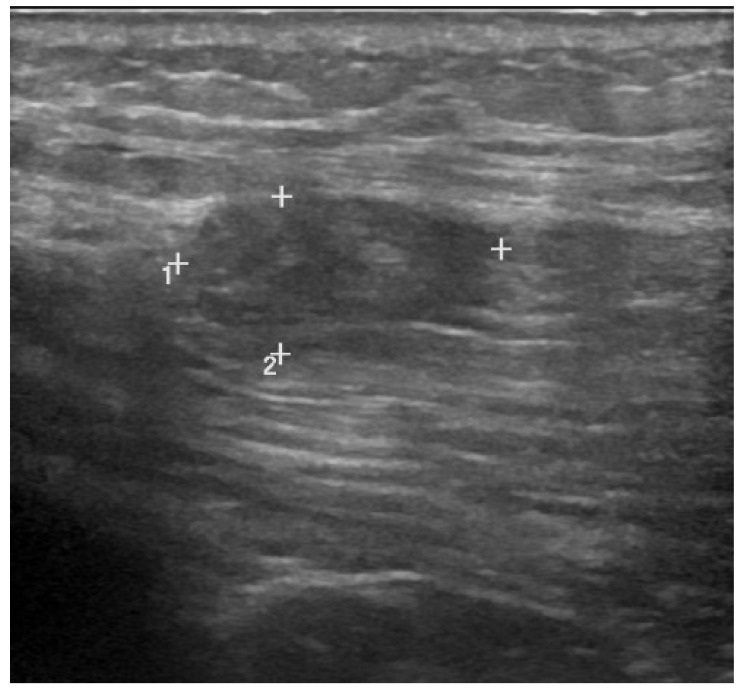
Grade 2 injury of the gluteus medius with interruption of muscle fibers and formation of hypoechoic local hematoma.

Muscle lesions show different US appearance depending on the type, extent and anatomical site involved. Following a blunt trauma of mild intensity, the capillaries and muscle fibers break causing interstitial hemorrhage that appears as a region of hyperechogenicity with poorly defined margins. In case of high intensity blunt trauma, an intramuscular hematoma will develop appearing to the US as a variable echogenicity zone (Figure 3) [11,24], depending on the time of the lesion. In the acute phase (24–48 h) the hematoma undergoes solidification appearing hyperechoic, due to its corpuscular component, compared to the surrounding tissue. In the next phase (48–72 h) it will undergo colliquation and progressive resorption, appearing as an iso-hypoechoic fluid zone. In the later stages, internal levels and debrides may be found in the hematoma fluid, and a focal scar may form as it undergoes resorption [25]. At the power/color Doppler it will be possible to observe a zone of hyperemia around the lesion due to the formation of granulation tissue representing the beginning of the reparative mechanisms [24]. In some cases, the rupture of the muscle fascia can occur with consequent herniation of the muscle in the interfascial space or in the subcutaneous tissue. This lesion can be seen, especially with dynamic US, as a muscle mass that emerges from the fascial defect during contraction [26]. A potential consequence of high-energy trauma is a Morel–Lavallée lesion. This condition is caused by the detachment of the fascia from the overlying subcutaneous tissue with a consequent accumulation of hemolymphatic fluid that can be easily identified through US [27].

Peetrons et al. graded US features of indirect muscle injury [28]. In grade 1, US images may be negative or show minimal signs of lesion. A poorly defined area of hypercogenicity can be found at the site of the lesion, commonly involving MTJ. Furthermore, a focal interruption in the muscle fibers can be found, affecting less than 5% of the cross-sectional area of the muscle belly, represented by a well-defined anechoic or hypoechoic zone. In this area, a small hematoma might be observed. Grade 2 injuries include partial lacerations and are identified by the presence of an area of interruption of the muscle fibers larger than 5% but lower than 100% of the cross-sectional area of the affected muscle. This condition is characterized by the presence of a large hematoma of variable echogenicity depending on the time of the lesion. Grade 3 injuries identify a total muscle tear characterized by complete interruption of the muscle fibers with different degrees of retraction of the lesion stumps and the formation of a large hematoma.

In all degrees of injury, we can observe perifascial fluid [9,24].

## 6. Classification and Grading of Muscle Injury

Despite the fact that muscle injuries are the most common sport-related trauma, there is a lack of consensus about their classification [29]. From the 1960s these lesions were classified according to clinical signs and pathophysiologic mechanisms. In 1966 Rachun proposed a three-grade classification based on clinical signs and symptoms, such as pain, swelling, loss of function, grade of disability [30]. Later, Wise focused on differences between normal and injured muscle circumferences, pain severity and loss of strength following contraction, and muscle spasm [31]. From the 1980s, with the development of US and MRI, classifications were modified taking into account imaging findings [32]. Lee et al. graded muscle injuries based on the tear extension and the percentage of function loss [33]. Schneider-Kolsky et al. emphasized functional aspects of muscle tears, considering ROM limitations to distinguish the various grades of lesions [34].

A new classification of muscle injuries, which derives from the previous grading systems, has recently been introduced (Table 1):

- Grade I: muscle injury with low disability, localized pain, small hemorrhage and swelling with mild ROM limitation (<10°);
- Grade II: moderate disability, pain and swelling, loss of function between 5% and 50% and moderate ROM limitation (10–25°);
- Grade III: muscle rupture with severe disability and pain, loss of function more than 50% and severe ROM limitation (up to 25°) [29].

Based on US appearance of muscle lesions, several authors have proposed different grading systems (Table 2). Takebayashi et al. and Peetrons classified muscle lesions according to the percentage of cross-sectional area of muscle involved [28,35]. In 2004 Lee et al. introduced other US findings, such as hypervascularity around damaged fibers and possible detachment of adjacent fascia, describing several types of muscle injuries [33]. In 2012, Chan et al. took into consideration the same US findings but introduced other additional features, i.e., the site of lesion, such as the proximal MTJ, muscle belly or distal MTJ and the specific part of muscle involved, such as intramuscular, myofascial, myotendinous [36].

In 2012, a consensus meeting endorsed by International Olympic Committee (IOC) and Union of European Football Associations (UEFA), proposed a comprehensive classification system, the “Munich Muscle Injury Classification” considering the mechanism of trauma [37]. According to this classification, direct injuries are divided into lacerations and contusions, both caused by blunt external force, with lacerations characterized by muscle rupture. Indirect injuries are divided into structural injuries and functional disorders. Structural injuries, unlike functional, show an anatomically evident lesion and need longer lay-off times [37,38].

Functional disorders are divided in overexertion-related muscle disorder (Type 1) and Neuromuscular disorder (Type 2). These lesions do not present any findings on US and MRI, although sometimes oedema could be observed.

Type 1 functional disorders include:

- Type 1A—*Fatigue-induced muscle disorder*, mostly caused by change in playing surface, is characterized by focal increased “muscle tightness” and dull pain;
- Type 1B—*Delayed Onset Muscle Soreness (DOMS)*, that is a more generalized dull pain caused by decelerations during eccentric contractions. It peaks within 24–72 h after activity.

Type 2 functional disorders include:

- Type 2A—*Spine-related neuromuscular muscle disorder*, that is a focal increase of muscle tone caused by structural or functional spinal disorder;
- Type 2B—*Muscle-related neuromuscular muscle disorder*, characterized by increased muscle firmness and cramp-like sensation, due to neuromuscular disorder.

Structural injuries are divided in Partial muscle tear (Type 3) and Complete muscle tear (Type 4) and are characterized by the following findings on US and MRI:

- Type 3A—*Minor partial muscle tear* involves less than a muscle fascicle and is characterized by localized pain and absence of visible hematoma;
- Type 3B—*Moderate partial muscle tear* involves more than a muscle fascicle but not all muscle belly, with palpable defect painful to touch, and visible hematoma;
- Type 3C—*Subtotal/Total muscle tear or tendinous avulsion* involves up to 90% of muscle belly, is characterized by severe pain, immediate functional impairment, and muscle retraction in case of avulsion [37,38].

In 2013 the “Italian Society of Muscles, Ligaments and Tendons” (ISMuLT) proposed guidelines about the management of athletes after muscle injury and added some items to the Munich Classification. Authors divided indirect injuries into structural and not structural, replacing the taxonomy “structural injuries” and “functional disorders”. Moreover, they added the prognosis of all types of muscle injury in terms of lay-off time before RTP and added the anatomical site of structural injuries, differentiating proximal (P), middle (M) and distal (D) muscle injuries. For example, considering the same severity of the lesion, a distal triceps surae injury is worse than proximal or middle ones, as well as a proximal lesion of hamstrings and rectus femoris is worse than middle or distal ones. Finally, authors divided contusions, considered as direct injuries, in three grades according to the ROM limitation [39].

It should be underlined that in some grading systems the disability and the loss of function are not objectively defined through validated rating scales.

## 7. Healing Process and Prognosis of Muscle Injury

After injury, muscle tissue undergoes a repair process which schematically includes three phases:

- Destructive phase: it occurs immediately after the trauma and is characterized by the necrosis of the muscle fibers, the development of an inflammatory process and the formation of a local hematoma;
- Reparative phase: usually starts from the second day, it is characterized by the removal of cellular debris and necrotic tissue by macrophage cells; the local production of growth factors will promote the formation of a fibrous scar and the revascularization of the area. During this phase, the satellite cells may differentiate into myoblasts and can partly drive the regeneration of muscle tissue;
- Remodeling phase: with the reorganization of the fibrous scar and the maturation of regenerated myofibrils, a progressive recovery of the functional capacity of the muscle can be observed [15].

**Figure 4:**
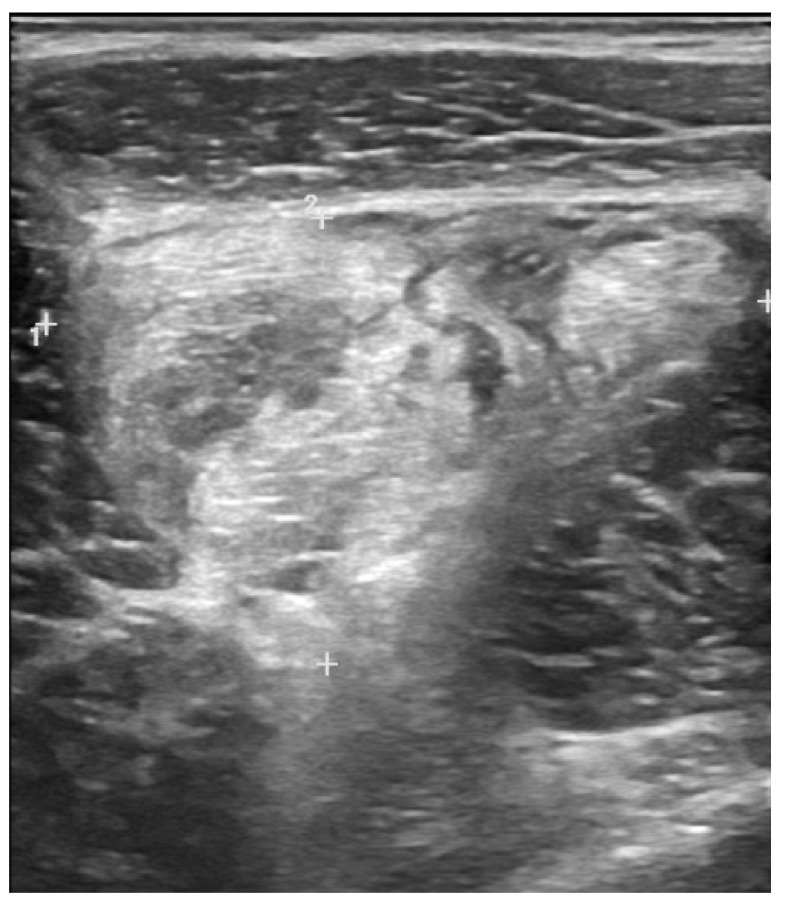
Grade 2 injury of the vastus medialis with hyperechoic healing fibrotic tissue.

Depending on the type and extent of the lesion, US imaging can provide useful information about muscle healing. In low grade muscle injury (grade 1) the reparative process appears as an increase in the echogenicity of the lesion area, with a progressive reduction of its extension. Higher grade lesions are characterized by the formation of a hematoma. During the reparative process, hematoma undergoes liquefaction resulting hypoechoic, with progressive resorption and reduction of its extension. Lesion margins will be hyperechoic and echogenic material inside the lesion, representing the deposition of scar tissue, will be observed [28] (Figure 4).

The site of scar formation should be properly evaluated, especially in case of abnormal symptoms persistence. This area has a greater collagen component and lower elasticity than normal muscle tissue, representing a site at higher risk for recurrent injuries [40]. Moreover, a dynamic US assessment during concentric contraction of the affected muscle is useful for proper identification of the lesion margins and the persistence of fiber disruption over the healing time [9].

An additional tool for healing evaluation of muscle injuries is the elastosonography. This technique allows real-time imaging of the tissue’s elasticity and is based on the principle that the compression of a tissue produces a strain or a displacement, which will be higher in soft tissue [41]. Normal muscle tissue shows a characteristic pattern on elastosonography, described as a heterogeneous mosaic of red, blue, and green colors [42]. The elastosonography findings following muscle injury will show increased elasticity (i.e., increased red color) in the site of the lesion attributable to the formation of the hematoma [43]. Later with the progressive resorption of the hematoma and the development of the scar, a blue area indicating the loss of elasticity will appear. Lesions with worse prognosis tend to show an area of reduced elasticity that extends beyond the US zone of the scar [44,45,46].

Few studies evaluated the prognostic value of US imaging in sport-related muscle injury. A longitudinal study of acute hamstring lesions assessed by MRI and US, examined the correlation between diagnostic findings and recovery time of the players (i.e., RTP). The authors found that the extent of the lesion, defined as cross-sectional area, and the presence of intramuscular hematoma, evaluated by US, are significantly correlated with prolonged RTP time [47]. Conversely, another study investigating the prognostic value of US in hamstrings injury in soccer players did not find any association between US findings, including the size of the lesion area and the RTP time [48]. Renoux et al. examining the role of connective tissue in muscle injuries, reported longer RTP times in elite athletes with connective tissue involvement at US imaging [49].

## 8. Complications of Muscle Injuries and Atypical Lesions

### 8.1. Myositis Ossificans

Myositis ossificans is a heterotopic ossification of muscle tissue usually secondary to trauma, which is found in up to 50% of cases [24] with higher prevalence in young adults practicing contact sports [50]. It appears as a new space-occupying mass in the muscle tissue that may be painful. It seems to be due to metaplasia of intramuscular connective tissue which result in local ossification [51]. This process takes 2 to 6 weeks before being visible to X-ray, therefore US is useful in the early evaluation of myositis ossificans. Different US findings can be found depending on the stages of the lesion. In the initial stages, myositis ossificans appears hyperechoic in the center of the muscle lesion with a hypoechoic periphery. Then an external hypoechoic zone with an increased Doppler activity develops, along with a hyperechoic intermediate zone and a hypoechoic central zone. In the later phase, calcification will begin from the periphery of the muscle lesion with the typical egg-shell aspect that will appear progressively more hyperechoic and reflective, with a posterior acoustic shadow, as the process progresses [52]. In case of doubt, it is advisable to carry out second level examinations such as computed tomography (CT) to better identify the calcified component of the lesion and differentiate it from other conditions like sarcomas or abscess [24].

### 8.2. Muscle Hernia

Muscle hernia is a rare condition characterized by the herniation of healthy tissue through an area of disruption of the lining muscle fascia (epimysium) following direct trauma [11], particularly in the lower limbs (i.e., the anterior tibialis is the most affected muscle) [53]. It presents as a chronic mass that can be painful and most evident during contraction. Ultrasound shows healthy muscle tissue protruding from a fascial gap more visible with dynamic evaluation. Moreover, a slight pressure of the probe on the lesion reduces it through the hernial path [54].

### 8.3. Compartment Syndrome

Compartment syndrome is a painful condition due to the increased pressure in a narrow fascial space that leads to compression and collapse of the capillary network in the affected compartment, with consequent ischemic tissue injury. Tissue damage is attributable to the formation of abundant intramuscular oedema or a large hematoma which increase the pressure in the closed compartment [24]. This syndrome affects the anterior, deep posterior, lateral, and superficial posterior muscle compartments of the lower limbs more often [55]. It presents as chronic and recurrent well localized pain, induced by physical activity [56]. Ultrasound can be useful to highlight lesions that can increase local pressure such as hematomas. Therefore, it can help in the differential diagnosis with other painful lesions such as venous thrombosis or arterial occlusion [57]. Ultrasound findings specific to compartment syndrome are often difficult to visualize and include increased muscle reflectivity, fascial bowing, loss of the fascicular aspect of the muscle, complete loss of central aponeurosis of the muscle in advanced stages associated with signs of diffuse muscle damage and rhabdomyolysis [58].

### 8.4. Muscle Atrophy

Muscle atrophy is the progressive degeneration of muscle tissue with loss of its function. It is generally consequent to complete rupture of muscle belly not adequately treated or following peripheral nerve injury, and it is an infrequent complication of sport-related injury [2]. Upon US examination, atrophy will appear as a progressive fat replacement of the muscle tissue (i.e., more echogenic than normal) starting from the central tendon of the muscle affected or from the MTJ. Later there will be a progressive thinning of the muscle with poor vascularization assessed by the Doppler [28].

## 9. Rehabilitation and Interventional Therapy

Appropriate management of sport-related muscle injury is based on a comprehensive personalized treatment, that include therapeutic exercise, physical therapies, and interventional approaches. Therapeutic exercise, starting from the 2nd day, consists of stretching techniques of the muscle group involved followed by isometric muscle strengthening according to the clinical and US findings. From the 4th to the 8th week, it will be possible to start functional training with muscle strengthening aimed to achieving pre-injury strength level [59]. The use of non-steroidal anti-inflammatory drugs (NSAIDs) is not recommended because they can alter the healing process by interfering with the synthesis of prostaglandins that are involved in early phases of tissue healing [60].

**Figure 5:**
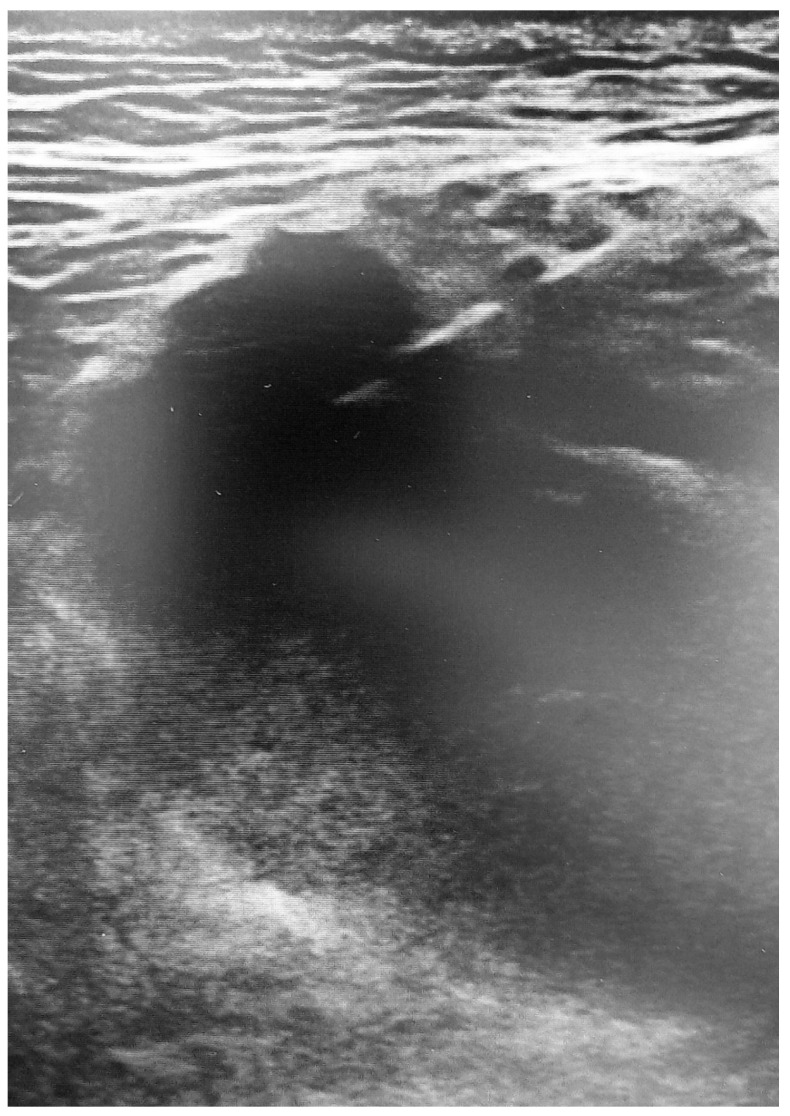
Anechoic fluid collection (large hematoma) drainage by hyperechoic needle (direct in-plane technique).

One of the most used interventional procedures is hematoma aspiration. It is particularly indicated in chronic hematomas that do not resolve after about 2 weeks, or in case of intense painful lesions or to accelerate the recovery, especially of elite athletes [61]. Aspiration of chronic hematomas might avoid complications such as calcifications, cyst formation, compression of nerve structures, compartment syndrome [28,62]. To successfully perform this procedure, it is mandatory to evaluate that the hematoma is in the liquid phase. An US-guided in-plane or out-of-plane aspiration can be performed once the precise location and extent of the hematoma have been identified. A standard 10 mL syringe with an 18–20 G needle can be used, taking care to maintain sterility during the procedure to avoid infections (Figure 5) [61].

Following the procedure, a tight elastic bandage should be kept in place to prevent the recurrence of the hematoma [63]. In this latter case, it may be useful to inject low doses of corticosteroids following drainage [64]. Another potentially useful interventional method in muscle injuries is the in-situ injection of Platelet-Rich Plasma (PRP) [24]. The rationale for the use of this tool lies in the ability of platelets to release growth factors that can promote tissue regeneration, myogenesis and angiogenesis. In this way, faster healing of the injured tissue may be achieved [65]. There are different formulations of PRP, depending on their production process: leukocyte-poor PRP (pure PRP, P-PRP) and leukocyte PRP (L-PRP) [66]. Both formulations are used in sports medicine in the form of a liquid or gel solution, with the latter having higher concentration of platelets. The dosage and timing of administration are variable. According to Orlandi et al., from 2 to 10 mL of PRP, and from one to three total injections, might be effective depending on the degree and extent of the lesion [61]. Several in vitro studies support the potential benefits of PRP in the treatment of muscle injuries [67,68], even if clinical studies reported conflicting results about the effectiveness of this procedure [69,70,71,72,73]. Setayesh et al. in a recent review suggest that these uncertainties are probably attributable to the great heterogeneity in PRP formulations and treatment protocols [74].

## 10. Strengths and Limitations of US Imaging in Sport-Related Muscle Injury

US imaging is frequently used in the evaluation of musculoskeletal pathologies as a first-line approach. Indeed, it is widely available, well tolerated, easy to use, fast, and cost-saving compared to MRI. Moreover, US imaging offers dynamic evaluations in real-time, being able to take advantage of the patient’s collaboration to better characterize the lesions. In particular, it allows the practitioner to visualize how and if the imaging findings change before and after an isometric contraction [9]. Therefore, US is useful in the clinical exam in identifying injured muscle and in differentiating between lesions with similar clinical features [2].

Despite the utility of US in the diagnosis and clinical management of muscle injury, it has some limitations. This technique is less sensitive than MRI in depiction of minor lesions, such as mild contusion and DOMS, because it is not able to visualize minimal oedema and lesions lacking fiber disruption. Regarding major trauma, the US and MRI demonstrate almost comparable sensitivity. In particular, there is complete agreement in the visualization of severe contusions [8]. On the other hand, both US and MRI prognostic values are uncertain.

## 11. Conclusions

Muscle injuries are responsible for loss of competition, long recovery times and risk of recurrent injury, both in professional and amateur athletes. An appropriate management is necessary for adequate healing to minimize RTP time, complications, and risk of recurrent injury. In this context, US may play a main role because it is fast and relatively cheap, allows serial evaluation of the healing process and dynamic muscle assessment. Dynamic evaluation of the anatomical and functional damage and the monitoring of healing progression of muscle injury represent key elements to better define the recovery in terms of both RTP time and sports performance, also driving the rehabilitation course of affected athletes.

## Figures and Tables

**Table 1:** Grading systems for muscle injuries.

	Rachun 1966 [30]	Wise 1977 [31]	Lee et al. 2004 [33]	Schneider-Kolsky et al. 2006 [34]	Grassi et al. 2016 [29]
**Grade I**	Localized pain, aggravated by movement, minor disability, mild swelling, ecchymosis, local tenderness, minimal haemorrhage.	Minimal pain to palpation, well localized.	Small tear, <5% loss of function	<10° ROM loss.	Minimal and localized pain, minimal hemorrage and swelling, mild ROM loss (<10°).
**Grade II**	Localized pain, aggravated by movement, moderate disability, moderate swelling, ecchymosis, local tenderness, stretching and tearing of fibers, without complete disruption.	Substantial pain to palpation, poorly localized; 6–12 mmdifference in circumference, develops within 12–24 h; <50% loss of ROM; pain on contraction with loss of power and disturbed gait.	Larger tear, 5–50% loss of function.	10–25° ROM loss.	Moderate pain, moderate swelling and disability, loss of function between 5% and 50% and moderate ROM loss (10–25°).
**Grade III**	Severe pain, and disability, severe swelling, ecchymosis, hematoma, palpable defect and loss of muscle function; muscle or tendon rupture.	Intractable pain to palpation, diffuse; >12 mm difference in circumference, develops rapidly within one hour; >50% loss of ROM; severe pain on contraction with almost total loss of power with flicker contractions and unable to bear weight.	Complete tear >50% loss of function.	>25° ROM loss.	Severe pain and disability, more of 50% of loss of function and severe ROM loss (up to 25°).

**Table 2:** US-based grading systems for muscle injuries.

	Takebayashi et al. 1995	Peetrons 2002	Lee et al. 2004	Chan et al. 2012
**Grade I**	“<20% cross-sectional area.”	“Minimal elongations with less than 5% of muscle involved.”	“Normal, or focal/general areas of increased echogenicity +/− peri-fascial fluid.”	“Normal appearance; focal or general increased echogenicity with no architectural distortion.”
**Grade II**	“20–50% cross-sectional area.”	“5–50% muscle involvement, partial muscle rupture, demonstrable hypo or an echoic gap, with “bell clapper” sign.”	“Discontinuity of muscle fibers in echogenic perimyseal striae; hypervascularity around disrupted muscle fibers; intramuscular fluid collection; partial detachment of adjacent fascia or aponeurosis.”	“Discontinuous muscle fibers; disruption site is hypervascularized and altered in echogenicity; no perimyseal striation adjacent to the MTJ.”
**Grade III**	“>50% cross-sectional area.”	“Complete tear of muscle or fascia, with extravasation of collection away from injured part of muscle.”	“Complete myotendinous or osteotendinous avulsion; complete discontinuity of muscle fibers and associated hematoma; “bell clapper” sign.”	“Complete discontinuity of muscle fibers; hematoma and retraction of the muscle ends.”

## Data Availability

Not applicable.
